# Maternal smoking in pregnancy and blood pressure during childhood and adolescence: a meta-analysis

**DOI:** 10.1007/s00431-023-04836-1

**Published:** 2023-02-24

**Authors:** Nerea Mourino, Leonor Varela-Lema, Jasjit S. Ahluwalia, Julia Rey-Brandariz, Cristina Candal-Pedreira, Alberto Ruano-Ravina, Andrea Vila-Farinas, Andrés Torres, Mónica Pérez-Rios

**Affiliations:** 1 Department of Preventive Medicine and Public Health, University of Santiago de Compostela, Santiago de Compostela, Spain; 2 School of Public Health and Alpert School of Medicine, Brown University, Providence, RI USA; 3 Consortium for Biomedical Research in Epidemiology and Public health (CIBERESP), Madrid, Spain

**Keywords:** Tobacco, Pregnancy, Blood pressure, Meta-analysis, Cohort studies, Children, Adolescents

## Abstract

Arterial hypertension during childhood or adolescence is rising, and smoking during pregnancy may constitute a modifiable risk factor. This study aims to evaluate the effect of maternal smoking during pregnancy on diastolic (DBP) and systolic blood pressure (SBP) in childhood and adolescence. A bibliographic search was conducted in PubMed, Embase, and CENTRAL databases in March 2022. Meta-analysis was performed with the difference in mean-adjusted SBP/DBP of children and adolescents aged 3–17 years, according to maternal smoking/non-smoking in pregnancy. A random effects model was applied; a leave-one-out analysis and meta-analysis by subgroups were performed. A modified Newcastle–Ottawa scale was used to assess the quality of the studies. Evidence levels were rated using the GRADE system. Fifteen studies were included in the meta-analysis; all of them evaluated the mean-adjusted SBP difference in children or adolescents (*N* = 73,448), and 6 also that of DBP (*N* = 31,459). Results showed that maternal smoking during pregnancy significantly increased SBP (*β* = 0.31 mmHg 95% CI 0.14–0.49). A greater increase in mean-adjusted SBP was observed in those studies that completed the recruitment before 1990, were conducted in non-European countries, used standard mercury or manual sphygmomanometry, adjusted for birth weight, and were in the lowest quality subgroup. No significant association was found for DBP. The GRADE level of evidence was low for SBP and very low for DBP.

*Conclusion*: Smoking in pregnancy might increase SBP in childhood and adolescence. Due to the low level of evidence, solid inferences cannot be drawn about the clinical relevance of these findings.

**Table:** 

**What is Known:** *• AHT is the leading cause of premature death among adults worldwide.* *• Deleterious effects derived from SHS exposure on children's health have been documented since early 1970. To date, there are contradictory results about the effects of prenatal SHS exposure on children’s BP.*	
**What is New:** *• Smoking in pregnancy may increase SBP during childhood and adolescence.* *• Maternal smoking during pregnancy could have greater influence on their offspring's SBP than on DBP.*

**Supplementary Information:**

The online version contains supplementary material available at 10.1007/s00431-023-04836-1.

## Introduction

High blood pressure (BP) or arterial hypertension (AHT) in children, defined as systolic blood pressure (SBP) or diastolic blood pressure (DBP) equal to or higher than the 95th percentile, by sex, age, and height, up to age 16, or ≥ 130/85 mmHg for age 16 or older [1], is an increasingly frequent condition during childhood or adolescence [2]. It often tends to be asymptomatic, and is underdiagnosed by health professionals [3, 4]. The global prevalence of AHT in the general pediatric population (children aged 6 to 19 years) is estimated at around 4.0%, with 3.0% for systolic hypertension and 1.9% for diastolic hypertension [2]. Even so, the US National Health and Nutrition Examination Survey shows that 13.3% of American children have high BP [5].

Like many chronic diseases, the etiology of AHT is complex and is thought to develop as a consequence of an interaction between genetic predisposition and the environment, mediated in part by epigenetic factors. It has been shown that tobacco use during pregnancy can cause changes in the placenta, generating fetal alterations which are associated with health problems, both pre- and postnatal [6]. Tobacco increases the risk of ectopic pregnancy, rupture of membranes, fetal mortality, intrauterine growth restriction, premature birth, low birth weight, and sudden infant death syndrome [7, 8]. Similarly, it can affect the growth of pulmonary parenchyma and airways, increasing the risk of asthma and respiratory infections, as well as giving rise to other cerebral and cardiovascular alterations [9, 10]. Globally, 1.7% of women are estimated to smoke during pregnancy; however, prevalence stands at 8.1% in the European region or at 5.9% in the Region of the Americas [11].

Since Barker published a study on the influence of adverse intrauterine conditions on postnatal development of cardiovascular diseases [12], a great deal of research has been done on how exposure to maternal smoking in the fetal period might affect the development of AHT during childhood, taking into account the atherogenic effect of this teratogen [13]. AHT is the leading cause of premature death among adults worldwide [14], and current physiopathologic and epidemiologic evidence suggests that childhood AHT increases the risk of essential AHT, as well as cardiovascular events later in life [15].

The precise role played by smoking during pregnancy in development of AHT in childhood and adolescence has not yet been established, and controversy remains with mixed findings from studies [16, 17]. Two previous reviews with meta-analysis were identified, but most of the studies included were cross-sectional, and the cohort studies included had a short follow-up time [16, 18]. Furthermore, one of the reviews drew no distinction between maternal smoking during pregnancy and children’s exposure to environmental tobacco smoke (ETS) [16].

The aim of this study was to analyze current evidence by conducting a systematic review with meta-analysis of prospective cohort studies and to evaluate the association between maternal smoking in pregnancy and their offspring’s DBP and/or SBP during childhood or adolescence.

## Material and methods

We carried out a systematic review with meta-analysis, following the standard PRISMA (*p*referred *r*eporting *i*tems for *s*ystematic reviews and *m*eta-*a*nalyses) guidelines [19]. The systematic review protocol was registered in the PROSPERO database and updated in December 2022 (registration no. CRD42021247824). During the review process, we identified the need to modify the eligibility criteria relating to study design and participants’ target age range to improve the quality of the evidence. This justifies the updating of the initial protocol recorded in PROSPERO. Reassuringly, following the guidelines of the Cochrane Handbook, sensitivity analyses were further performed to identify if these particular decisions influenced our findings.

### Search strategy

In March 2022, we conducted a bibliographic search of the MEDLINE (PubMed), EMBASE, and CENTRAL databases, applying a pre-designed search strategy (Supplementary Table 1) drawn up by 3 expert reviewers in the matter. The search terms included both MeSH and free terms: “tobacco smoke pollution,” “smoking,” “tobacco smoking,” “cigarette smoking,” “pregnancy,” “hypertension,” “blood pressure,” “passive smoking,” “secondhand smoke,” “environmental tobacco smoke,” “smok*,” “maternal smoking,” “paternal smoking,” “arterial pressure,” “hypertension,” “systolic pressure,” “diastolic pressure,” and “mean blood pressure.” A manual review of the bibliographic references from included studies was performed to ensure the inclusion of all possible studies. No restrictions were applied in terms of country, study period, study design, or language.

### Eligibility criteria

The review covered studies that evaluated the association between maternal smoking in pregnancy and the BP of their offspring aged 3 through 17 years. We included all studies published in peer-reviewed journals that met the following PECOS question: “Among children or adolescents, what is the effect of maternal smoking during pregnancy on their mean-adjusted DBP and/or SBP, compared to those whose mothers did not smoke?”. The PECOS framework focuses on population, exposure, comparator, outcome and study design: Population: Pregnant mothers and their offspring aged 3 through 17 years; exposure: Tobacco use during pregnancy; comparator: Non-tobacco use during pregnancy; outcome: Difference in children’s or adolescents’ mean-adjusted DBP and/or SBP (in mmHg); Study design: Prospective cohort studies that provided the necessary data to calculate mean-adjusted differences and their 95% confidence intervals (95% CIs).

The studies with the following characteristics were excluded: studies that did not provide sufficient data to calculate mean-adjusted BP differences and their 95% CIs; studies including women who consumed exclusively e-cigarettes or other tobacco products; studies that reported combined results (women’s use of other tobacco products in addition to cigarettes); studies that evaluated exposure to ETS during pregnancy, not exclusively due to maternal smoking; studies that estimated mean DBP and/or SBP without adjustment for possible covariates; studies that included population under 3 years old or over 17 years old; studies that included exclusively female or male population since we intended to examine maternal smoking effects jointly in both sexes; and studies that included smoking children and/or adolescents. Furthermore, we excluded studies published in a language other than Spanish, English, or Portuguese, and communications to congresses, letters to the editor, opinion articles, narrative reviews, case–control studies, cross-sectional studies, case series, simulation studies, and studies which had been withdrawn. Although cross-sectional studies were identified, they were not included in the main meta-analysis to avoid misleading results.

### Selection of papers and extraction of data

After eliminating duplicated papers, 3 researchers screened the titles and abstract of all the papers yielded by the search. Each researcher evaluated eligibility separately on the basis of the title and abstract. In the case of papers considered potentially relevant, the full text was read to ensure that they fulfilled the inclusion/exclusion criteria. Differences of opinion about the inclusion or exclusion of any given paper were settled by consensus of the 3 reviewers. Where different papers based on the same study were identified, we included those that had the largest sample size and with the most up-to-date data.

Where the outcome variable was adjusted for different confounding variables, we selected the model with the best fit. In the case of studies that exclusively provided mean-adjusted DBP and/or SBP of children and/or adolescents according to maternal smoking, we used the EPIDAT program to calculate the coefficient of the difference between means along with the 95% CI.

To extract the relevant information from each paper, a data extraction sheet was designed in Microsoft Excel. The data were manually extracted by 2 of the authors, and both sets of extractions were then reviewed by a third. Differences of opinion were discussed and settled by consensus. From each paper, data were extracted on [1] study design; [2] author and year of publication of the study; [3] period of recruitment of pregnant women; [4] country of study; [5] data source; [6] age of children in whom BP was evaluated; [7] sample size; [8] definition of maternal tobacco use in pregnancy; [9] number of BP measures; [10] BP measurement method, i.e., oscillometry or digital sphygmomanometry and standard mercury or manual sphygmomanometry; [11] difference in mean-adjusted SBP/DBP (in mmHg) according to maternal smoking in pregnancy, along with the 95% CI; and [12] adjustment for confounding variables.

### Assessment of quality and level of evidence

The AMSTAR 2 tool was used to score the systematic review [20]. The quality of the studies included was evaluated using an adaptation of the Newcastle–Ottawa scale [21]. Two researchers screened each study separately by reference to the representativeness of the sample, sample size, ascertainment of the exposure to maternal smoking, number of BP measurements obtained, adjustment for covariates, and statistical test. Supplementary Table 2 shows detailed information on the criteria and the number of points assigned to the studies according to each item. Studies were scored from 0 to 10 by each researcher, with the final score being reached by agreement. In the event of any difference of opinion, a third researcher was consulted. Studies that obtained a score of < 5 points were rated as poor quality, those with a score of 5–6 points as moderate quality, and those with a score of ≥ 7 points as high quality. While no studies were excluded on the basis of the evaluation of risk of bias, a sensitivity analysis was nonetheless performed with the higher quality studies.

Evidence levels were rated using the GRADE (*G*rading of *R*ecommendations, *A*ssessment, *D*evelopment and *E*valuation) system. The GRADE system allows for classification of evidence into four grades of evidence (high quality, moderate, low, and very low) attending to the risk of bias, inconsistency, uncertainty, inaccuracy, publication bias, and other considerations [22].

### Data analysis

To perform the meta-analysis, we calculated the difference in mean-adjusted SBP/DBP of children or adolescents, according to whether their mothers had or had not been smokers during pregnancy. A random effects model was applied, and differentiated analyses were performed for DBP and/or SBP, using the study’s covariate-adjusted results.

Inter-study heterogeneity was evaluated using the *p*-value of the Cochrane *Q* test and the *I*^2^ statistic. A *p-*value < 0.1 indicates the presence of heterogeneity, with such heterogeneity being considered substantial if *I*^2^ > 50% [23–25]. Presence of publication bias was analyzed using a funnel plot, Egger’s regression test for funnel plot asymmetry, and Begg’s test [26, 27]. In cases where the *p-*value is < 0.1, risk of publication bias was deemed to exist.

We performed both a leave-one-out analysis to ascertain the influence of each of the studies, sensitivity analysis including cross-sectional studies and one prospective cohort study with 18-year-old males, and the following meta-analyses by subgroups: (1) study quality (low vs. medium/high); (2) studies that adjusted for children’s birth weight (yes vs. no); (3) recruitment period (1958–1989; 1990–2000; 2001–2007); (4) continent (European or non-European); (5) BP measurement method (digital sphygmomanometry or oscillometry vs. standard mercury or manual sphygmomanometry); and (6) age group (3–6.5 years vs. 7–15 years). All analyses were performed using the STATA statistical analysis software program v17.

## Results

### Search results

**Fig. 1:**
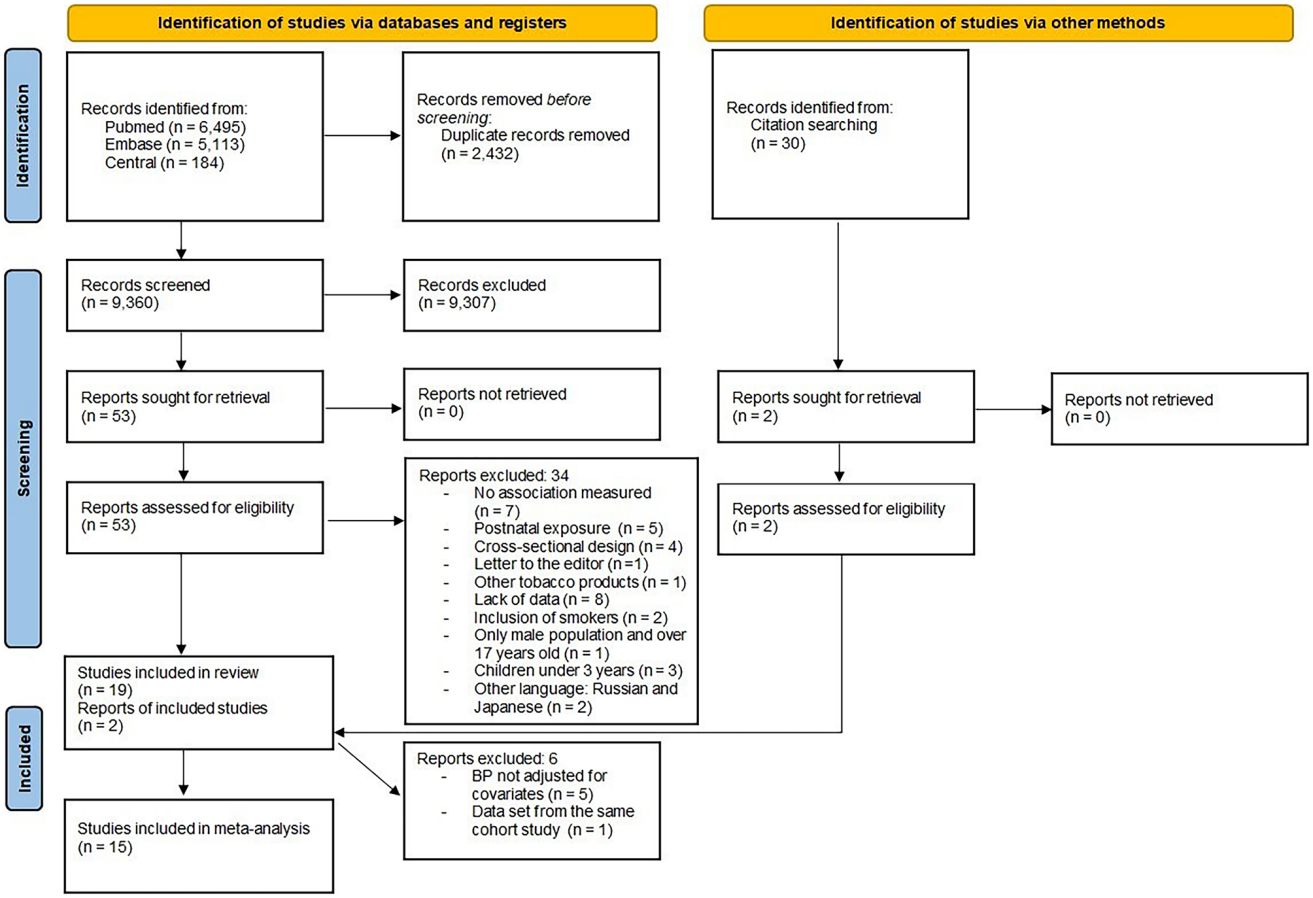
Flowchart of studies selected for systematic review and meta-analysis in accordance with the PRISMA 2020 guidelines

The search yielded a total of 12,035 papers; 21 studies fulfilled the inclusion criteria and 15 were included in the meta-analysis (Fig. 1). Supplementary Table 3 shows the characteristics of those potentially relevant studies excluded from the systematic review after reading the full text (*n* = 34), including cross-sectional studies [28–31] and a prospective cohort study with 18-year-old males [32].

### Characteristics of the studies included

All of the 15 studies included in the meta-analysis evaluated the difference in the mean-adjusted SBP of children and/or adolescents, aged 3 to 15 years, according to maternal use/non-use of tobacco (*N* = 73,448), and 6 also evaluated the difference in the mean-adjusted DBP (*N* = 31,459) (Table 1).

**Table 1:** Main characteristics of cohort studies

**Author, year (reference)**	**Recruitment period**	**Country**	**Source**	**Age**^**c**^ **(years)**	**N**	**Definition of maternal smoking (pregnancy)**	**BP measures (method)**^**a**^	**Adjusted (SBP/DBP)**
Law et al. 1991 [33]	1984–1985	UK	Hospital	4–4.5	405	Daily cigarette use	3 × SBP (1)	Yes
Morley et al.^d^ 1995 [42]	Not shown	UK	Hospital	7.5–8	618	Daily cigarette use	3 × SBP + DBP (1) + (2)^b^	Yes/yes
Williams and Poulton 1999 [43]	1972–1973	New Zealand	Hospital	9	795	Use during pregnancy (yes/no)	3 × SBP + DBP (2)	Yes/no
Bergel et al.^d^ 2000 [46]	1987–1990	Argentina	Clinical profile	5–9	518	Use during pregnancy (yes/no)	3 × SBP + DBP (2)	Yes/no
Blake et al.^*^ 2000 [49]	1989–1992	Australia	Hospital	6	702	Use during pregnancy (yes/never)	2 × SBP + DBP (1)	No/no
Lawlor et al. 2004 [44]	1981–1984	Australia	Hospital	5	3299	Use during pregnancy (yes/never)	3 × SBP (1)	Yes
Oken et al. 2005 [47]	1999–2002	USA	Clinical profile	3	689	Use during beginning of pregnancy	5 × SBP (1)	Yes
Brion et al. 2007 [61]	1991–1992	UK	Population	7	6509	Use in any trimester (yes/no)	2 × SBP + DBP (1)	Yes/yes
Laura et al.^*^ 2010 [64]	1993	Brazil	Population	11	4452	Use during pregnancy (yes/no)	2 × SBP + DBP (1)	No/no
Wen et al. 2011 [36]	1959–1965	USA	Hospital	7	30,461	Use during pregnancy (never, moderate, intense)	1 × SBP (2)	Yes
Ayer et al. 2011 [48]	1997–1999	Australia	Hospital	8	405	Use during pregnancy (yes/no)	3 × SBP + DBP (1)	Yes/no
Belfort et al.^2^ 2012 [45]	1984–1985	USA	Hospital	6.5	694	Use during pregnancy (yes/no)	3 × SBP (1) + (2)^b^	Yes
Leary et al. 2013 [62]	1991–1997	UK	Population	15	4723	Use during pregnancy (yes/no)	2 × SBP (1)	Yes
Taal et al. 2013 [34]	2002–2006	The Netherlands	Population	6	5447	Continued smoking during pregnancy	4 × SBP + DBP (1)	Yes/yes
Van den Berg et al. 2013 [41]	2003–2004	The Netherlands	Hospital	5–6	3024	Use during pregnancy (yes/no)	2 or 3 × SBP + DBP (1)	Yes/yes
Yang et al. 2013 [63]	1996–1997	Bielorrusia	Hospital	6.5	12,196	Use during pregnancy (yes/no)	2 × SBP + DBP (1)	Yes/yes
Rauschert et al.^*^ 2019 [73]	1989–1999	Australia	Hospital	17	740	Use during the 18th and 34th week of gestation (yes/no)	^fn^SBP + DBP (not shown)	No/no
Xie et al. 2020 [60]	GECKO/ABCD: 2003–2007	The Netherlands	Population	5–6	1613/2052^e^	Use during pregnancy (yes/no)	3 × SBP + DBP (1)	Yes/yes
De Smidt et al.^*^ 2021 [74]	2006–2017	South Africa	Hospital	5	500	Use during pregnancy (yes/no)	3 × SBP + DBP (1)	No/no
Kok Grouleff et al.^*^ 2021 [75]	2019–2020	Greenland	Hospital	3.5–5.5	76	Use during pregnancy (yes/no)	^nf^SBP + DBP (not shown)	No/no
Cajachagua-Torres et al.^*^ 2021 [76]	2002–2006	The Netherlands	Population	10	4792	Continued smoking during pregnancy	3 × SBP + DBP (1)	No/no

*N *number of observations, *UK *United Kingdom, *USA *United States of America, *SBP *systolic blood pressure, *DBP *diastolic blood pressure

^*^Studies excluded from the meta-analysis

^a^(1) oscillometry or digital sphygmomanometry; (2) standard mercury or manual sphygmomanometry

^b^Used both methods to measure BP; however, the authors specified that most of the readings were taken with oscillometry

^c^It refers to the minimum and maximum ages (in years) of those children or adolescents included in the study

^d^Data sourced from clinical trials

^e^Sample size of 2 cohorts, GEYCKO and ABCD, respectively

^f^Not specified

Most studies recruited pregnant women before or during the 1990s (*n* = 12), were conducted in non-European continental areas (*n* = 12), and used oscillometry to measure BP (*n* = 12). With the exception of one study, all of them obtained at least 2 BP readings. Six studies were excluded from the meta-analysis because they did not adjust BP for covariates, and because the data came from the same study (Generation R) as another paper with a larger sample size (Table 1).

Maternal smoking during pregnancy was measured using self-reported questionnaires. One study evaluated use with determination of cotinine in maternal serum during pregnancy and in cord blood (Table 1).

Adjustment variables, related both to the mother, offspring, father/partner, and BP measurement, differed among the studies, yet most adjusted for sex, age, height, weight, and socioeconomic status and/or educational level (Table 2).

**Table 2:** Adjustment variables of studies included in the meta-analysis

**Authors**	**Child/adolescent**	**Mother**	**Father/partner**	**BP measurement**
**Law et al.** [33]	Weight at age 4 years	SBP		
**Morley et al.** [42]	Birth weight and current age and weight	Socioeconomic level, single or multiple pregnancy		Measurement method
**Williams et al.** [43]	Sex, birth weight, current weight, height, and BMI	Height, single or multiple pregnancy, marital status, and socioeconomic level by educational level	Socioeconomic level by educational level	
**Bergel et al.** [46]	Sex, age, current height, and BMI	Calcium supplementation during pregnancy		
**Lawlor et al.** [44]	Sex, birth weight, current age, weight, and height	Age, BMI prior to pregnancy, order of birth of boy/girl, educational level, and income during pregnancy		
**Oken et al.** [47]	Sex, birth weight, current age, height, and BMI	Socioeconomic level by educational level and income, SBP in 3rd trimester, race/ethnicity, and parity		BP measurement conditions (order of readings, cuff size, arm position, and child’s activity)
**Brion et al.** [61]	Sex, current age, and BMI	Age at the birth, parity, height, BMI, breastfeeding, and socioeconomic level by social class and educational level	Age, height, BMI, socioeconomic level by social class, and educational level at child’s birth	
**Wen et al.** [36]	Sex and gestational age	Age at pregnancy, race/ethnicity, marital status, socioeconomic level, and parity		
**Ayer et al.** [48]	Current exposure to ETS at home			
**Belfort et al.** [45]	Sex, current age, and height	Age, educational level, race/ethnicity, and annual income in household		Measurement method, and child’s behavior during measurement
**Leary et al.** [62]	Sex, birth weight, age, current height, and BMI	Age, height, BMI prior to pregnancy, history of AHT, parity, socioeconomic level by educational level, and social class (job occupation)	Age, height, and BMI prior to child’s birth	Room temperature, time of day, and child not talking during measurement and cuff size
**Rob Taal et al.** [34]	Sex, gestational age, birth weight, current age, and BMI	Age, parity, educational level, race/ethnicity, BMI prior to pregnancy, BP, and breastfeeding		
**Van der Berg et al.** [41]	Sex, age, height, and race/ethnicity	Adequacy of income, and socioeconomic level by educational level		
**Yang et al.** [63]	Sex and age	Age, height, BMI, educational level, occupation, marital status at child’s birth, order of birth (no. of older siblings in household), alcohol consumption during pregnancy	Age, height, BMI, educational level, occupation, marital status at child’s birth, and tobacco use	
**Xie et al.** [60]	Sex, gestational age, birth weight, current age, height, and BMI, and early increase in BMI	Educational level, BMI prior to pregnancy, AHT, and breastfeeding		

*SBP *systolic blood pressure, *BMI *body mass index, *ETS *environmental tobacco smoke, *AHT *arterial hypertension

Regarding the sources of funding for the 15 studies included in the meta-analysis, most study authors indicated that studies received funding (*n* = 13), particularly, from universities, Medical Research Council and National Institutes of Health; of note, the authors from one study did not disclose information on financial support [33].

### Study quality

The systematic review was rated as high quality using the AMSTAR 2 tool, considering that it provides an accurate and comprehensive summary of the results of available studies addressing the question of interest.

In terms of the quality scores, 5 studies were rated as being high quality, 5 as moderate, and 5 as low quality when using a modified Newcastle–Ottawa scale (Supplementary Table 4). Most of the studies displayed classification bias.

### Results of the meta-analysis

**Fig. 2:**
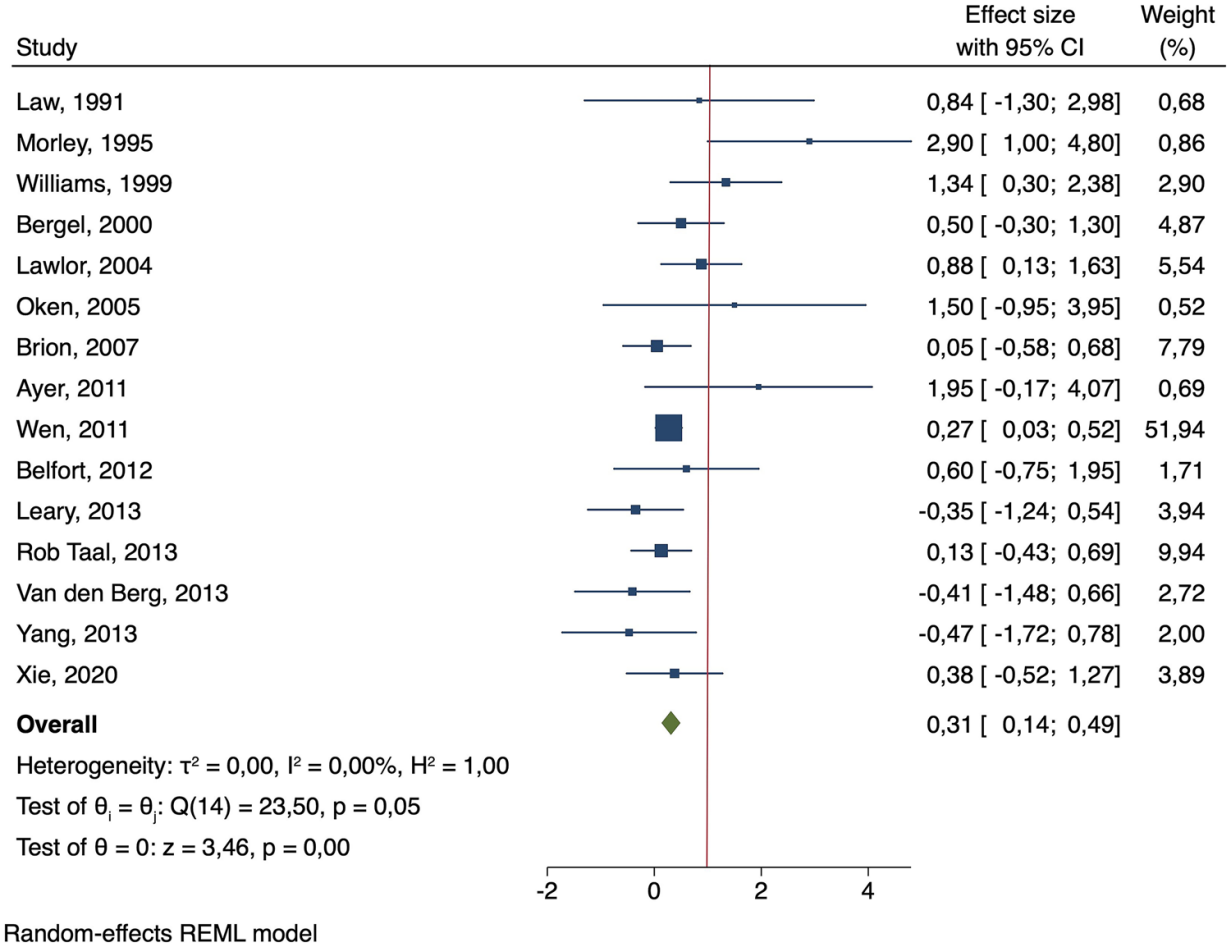
Forest plot of studies which measured difference in mean-adjusted systolic blood pressure(in mmHg) between children and adolescents exposed or not exposed to maternal smoking in pregnancy

**Fig. 3:**
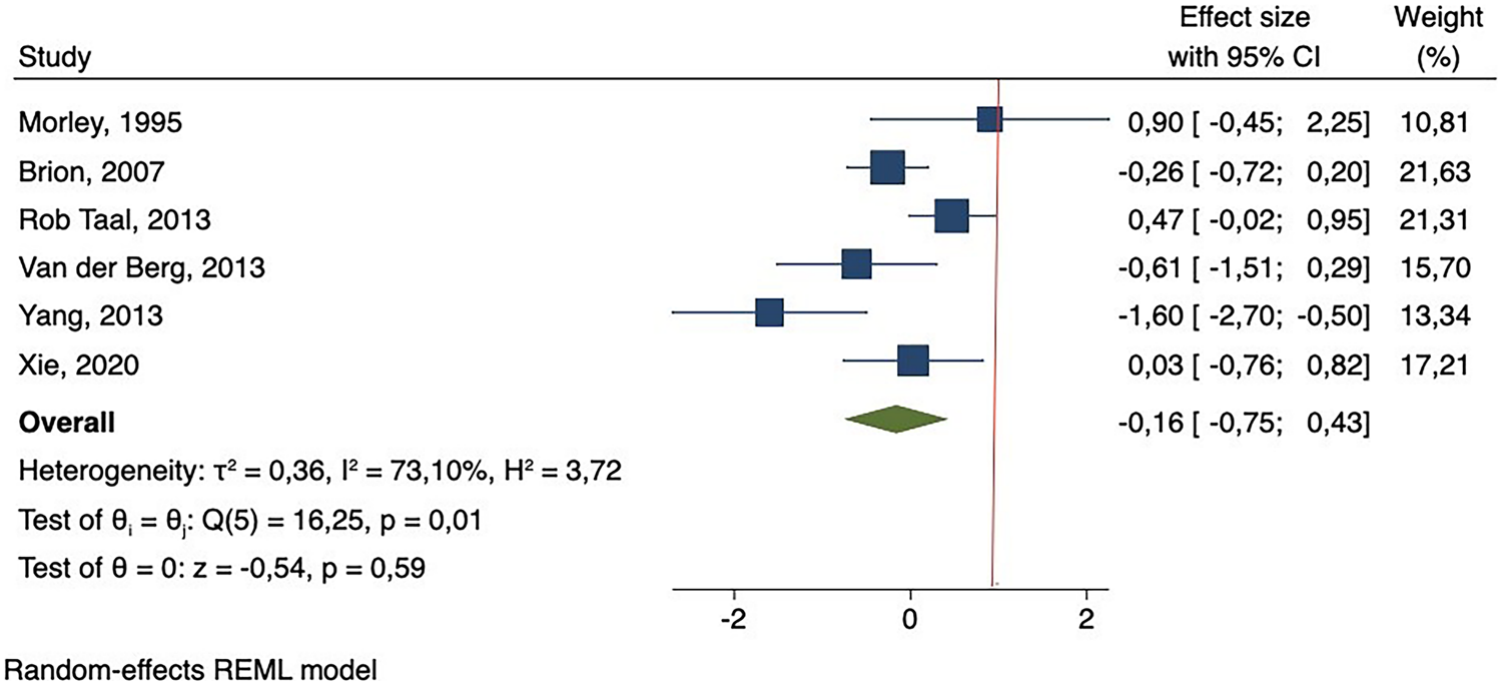
Forest plot of studies which measured difference in mean-adjusted diastolic blood pressure

Maternal smoking in pregnancy significantly increased SBP during childhood or adolescence (*β* = 0.31 mmHg; 95% CI: 0.14–0.49) (Fig. 2). The Cochrane *Q* test indicated that there might be inter-study heterogeneity (*p*-value < 0.1) but that such heterogeneity was not substantial (*I*^2^ = 0.00%). The leave-one-out analysis showed that none of the studies significantly modified the results (Supplementary Fig. 1). The funnel plot (Supplementary Fig. 2) and Egger’s test suggested that there might be publication bias (*p*-value < 0.1). No significant associations were found for DBP (*β* =  −0.16 mmHg; 95% CI: −0.75–0.43) and inter-study heterogeneity was high (*I*^2^ = 73.10% and *p*-value of the *Q* test < 0.1) (Fig. 3). None of the studies contributed substantially to reducing this heterogeneity (Supplementary Fig. 3). Supplementary Fig. 4 shows the funnel plot.

Findings from the meta-analysis including cross-sectional studies show similar results and great heterogeneity for both SBP and DBP (*β* = 0.37 mmHg; 95% CI: 0.03–0.70 [*I*^2^: 79.33%] and *β* =  −0.13 mmHg; 95% CI: −0.62–0.35 [*I*^2^: 59.76%], respectively] (Supplementary Figs. 5 and 6). Inter-study heterogeneity disappeared in the sensitivity analyses restricting to prospective cohort studies measuring SBP (Fig. 2). After including the prospective cohort study with 18-year-old males in the meta-analysis, similar results were found for both SBP and DBP (*β* = 0.26 mmHg; 95% CI: 0.13–0.38 [*I*^2^: 0.00%] and *β* =  −0.04 mmHg; 95% CI: −0.54–0.47 [*I*^2^: 82.77%], respectively) (Supplementary Figs. 7 and 8).

Regarding the meta-analyses conducted by subgroups, a greater increase in mean-adjusted SBP was observed in the subgroup of studies that completed the recruitment before 1990, were conducted in non-European continental areas, used standard mercury or manual sphygmomanometry, adjusted for birth weight, and were in the lowest quality subgroup. However, inter-study heterogeneity was substantial for some subgroups (Table 3). Subgroup analyses were not deemed for studies measuring DBP due to the small number (*n* = 6) and marked inter-study heterogeneity.

**Table 3:** Results of meta-analysis by subgroups

	***N***	**Adjusted SBP**		
		***β***	**95% CI**	***I***^**2**^** (%)**
**Overall**	15	0.31	0.14–0.49	0.00
**Subgroups**				
**Quality of the study**^**a**^				
Low	5	0.94	−0.09–1.97	59.99
Moderate-high	10	0.26	0.08–0.45	0.00
**Adjustment for birth weight**				
Model adjusted for birth weight	7	0.69	0.06–1.32	64.25
Model without adjustment for birth weight	8	0.25	0.04–0.45	0.00
**Recruitment period**				
1959–1989	6	0.87	0.28–1.47	57.21
1990–2000	5	0.10	−0.31–0.50	0.00
2001–2007	4	0.14	−0.28–0.57	0.00
**Continent**				
European	8	0.09	−0.22–0.40	0.00
Non-European	7	0.67	0.25–1.09	36.94
**Method for blood pressure measurement**^**b**^				
Oscillometry or digital sphygmomanometry	12	0.32	−0.04–0.68	30.04
Standard mercury or manual sphygmomanometry	5	0.81	0.15–1.47	43.01
**Age group**				
3–6.5 years	8	0.30	−0.07–0.67	9.96
7–15 years	7	0.62	−0.01–1.24	0.00

^a^Quality of the study was rated by applying a modified Newcastle–Ottawa scale. Studies that obtained a score of < 5 points were rated as low quality, those with a score of 5–6 points as moderate quality, and those with a score of 7 points or more as high quality

^b^Two studies (Belfort et al. and Morley et al.) measured BP with both oscillometry and manual sphygmomanometry

The GRADE level of evidence was low for SBP and very low for DBP (Supplementary Table 5).

## Discussion

These results show that smoking in pregnancy increases children’s and adolescent’s SBP, though the difference in adjusted-means is small (< 1 mmHg). With respect to DBP, no effect is in evidence, though the studies are very heterogeneous. Part of this heterogeneity could be due to some factors, including different adjustments for covariates and classification of maternal smoking, participants’ characteristics, or BP measurement method.

Importantly, the fact that maternal smoking during pregnancy was associated with SBP but not DBP could be due to effects on the arterial stiffness or pulse pressure, which mainly influences SBP, rather than effects on peripheral vascular resistance, which predominantly affects DBP [34]. Fetal growth retardation and arterial resistance adaptations resulting from exposure to maternal smoking could lead to altered elastin synthesis, which becomes more pronounced with age; gradual loss of elastin and replacement by collagen may result in reduced distensibility of the aorta and large arteries, leading to higher SBP [35].

The estimates are consistent with those of a previous one conducted in 2008 [17], which observed a higher increase in BP; however, it drew no distinction between SBP and DBP (0.62 mmHg 95% CI: 0.19–1.05) [17]. The results also coincide with those of another meta-analysis [16], which observed that passive exposure to tobacco (parental smoking or exposure to ETS from other smokers) significantly increased SBP (*β* = 0.26 mmHg 95% CI 0.12–0.39) but not DBP (*β* = 0.07 mmHg 95% CI −0.15–0.29). As with our analysis, the number of studies which evaluated DBP was limited to evaluate the possible causes of heterogeneity.

With the sole exception of one study [36], maternal smoking was measured with self-report, and most studies defined maternal smoking as “use during pregnancy: yes/no.” This definition is too broad and does not allow us to know the daily amount of tobacco cigarettes, the intensity, or the specific consumption according to the week of gestation. Although self-reported questionnaires have been widely used to evaluate prenatal exposure to ETS, and their validity is rated as high by some researchers [37], they could underestimate the prevalence of children’s exposure, due to pregnant women’s reticence to reveal their smoking status, social-desirability, or memory bias [38–40]. In this respect, whenever feasible, prenatal exposure should be measured with maternal biomarkers such as cotinine to provide a more accurate estimate.

Prior investigators found a dose-dependent association between maternal smoking during pregnancy and children’s or adolescents’ BP [16, 41]. Unfortunately, this could not be tested in this meta-analysis since just 2 studies recorded the daily cigarette consumption [33, 42].

Increased SBP was greater in the group of children and adolescents exposed to maternal tobacco smoke who participated in studies whose recruitment was conducted during 1959–1985 [33, 36, 42–45], compared to those with recruitment after 1985, and in that from non-European studies [36, 43–48], compared to European. These findings could be due to greater cigarette consumption by smoking mothers during pregnancy, as a consequence of lower awareness and social concern about the harmful effects for the fetus [49–52], worse diagnosis and treatment of gestational hypertension [35, 53], or lower prevalence and/or shorter duration of maternal breastfeeding [44, 54]. Alternatively, maternal socioeconomic status, parity and age at childbirth, and child’s BMI could also have influenced the results [28, 33, 41, 44, 54]; however, when examining the available data from the meta-analyzed studies, it was observed that the majority of the mothers were multiparous, were between 25 and 35 years old, and had average household income, and their children’s BMI ranged from 15 to 17 kg/m^2^.

The difference in mean-adjusted SBP was significant only in children and adolescents from studies in which BP was measured with standard mercury or manual sphygmomanometry [36, 42, 43, 45, 46]; however, it should be noted that these studies were mainly conducted before 1990 and in Non-European countries. Previous studies displayed discrepancies in terms of overestimation or underestimation of SBP according to the measurement method (oscillometry vs. standard mercury or manual sphygmomanometry) [55–57]. Accuracy of children’s BP reading could be influenced by the equipment (cuff size and calibration), subject (previous activity and activity during measurement, age, and height), setting and time of day of BP reading, and measurement technique (device, staff training, and experience) [3, 58].

We noted that the information provided in the studies included in the meta-analysis regarding staff training, cuff size, extremity used for the measurement, patient position, and environmental factors varied from one study to another. Of note, some studies did not follow a standard procedure and did not comply with the most recommended practices [59]; thus, one paper indicated that the BP measurement was obtained with the child in the supine position rather than seated [41]; two studies stated that the BP measurement was performed on the left arm rather than the right arm [33, 60], and some papers did not specify whether they used the appropriate cuff size for the child [36, 60].

The heterogeneity among the study participants could also affect BP estimates. Children’s age differed by studies [34, 41, 42, 44–47, 60–63]. After conducting the subgroup analysis by age group, we observed a higher increase in SBP among those studies including older children; however, statistical significance was not reached. Interestingly, 3 of the meta-analyzed studies [43, 62, 64] measured BP during puberty and observed that the association between mothers’ smoking in pregnancy and BP of their offspring did not vary according to whether measurements were obtained before or during initiation of puberty.

Some of the meta-analyzed studies excluded twins, children with congenital heart disease or kidney abnormalities, premature births, and newborns with low or high birth weight for gestational age, which could have underestimated the total effect of tobacco smoke exposure on child’s BP [33, 34, 36, 43, 57, 63]. The unique study that included exclusively premature children and children with low birth weight [45] reported no statistically significant increase in mean-adjusted SBP of children exposed to maternal tobacco smoke, compared to those unexposed. In the subgroup analysis, the difference in mean-adjusted SBP was greater in the group of studies that adjusted for birth weight with respect to those that did not. A previous study concluded that the direct effect of birth weight on children’s and adolescents’ BP could be overestimated, when taking into account the indirect effect of this variable on children’s height and BMI; in this case, all the studies which adjusted for birth weight, with the exception of 3 [34, 42, 44], also did so for children or adolescents’ height and BMI. Specifically, 8 studies adjusted BP for child’s height [41, 43–47, 62], and all but 5 studies [36, 41, 45, 48, 63], for child’s weight or BMI.

This meta-analysis has a series of strengths. First, we evaluated the differences in mean-adjusted BP (in mmHg) and not the probability of suffering from AHT, thereby reducing the risk of incorrect classification, in view of the different criteria for defining AHT in children and adolescents [3, 60]. Second, our inclusion criteria were strict and we focused on cohort studies with longitudinal measures of BP. Third, our results make it possible to examine the difference in SBP and DBP across almost 5 decades, considering adjustment for different covariates, for a total of 73,448 children and adolescents whose mothers smoked during pregnancy.

However, the overall quality of evidence was judged to be low for SBP and very low for DBP. The certainty in the evidence was downgraded due to the high risk of bias in the individual studies included for both outcomes. Several studies displayed bias related to the design and analysis such as selection, sample size, classification of prenatal exposure to maternal smoking, and confounding. Confounding bias was the most widely observed limitation in low- and moderate-quality studies due to failure to adjust for important variables which could have confounded the association, or due to adjustment for potential causal intermediates which could result in an underestimation of the total effect of maternal smoking during pregnancy on child’s BP, such as birth weight or gestational age. For example, we are aware that increase in child’s SBP could be influenced by current ETS exposure as some mothers could continue smoking but just one of the studies included in the meta-analysis adjusted BP for this covariate. Reassuringly, although no studies were excluded on the basis of the evaluation of risk of bias, a sensitivity analysis was performed considering the quality rating assigned to the individual studies according to the adapted Newcastle–Ottawa scale, and higher increase was observed in the subgroup of studies rated as being low quality but increase was just statistically significant for those moderate-high quality studies. We did not include gray literature, and therefore, this could have contributed to the risk of publication bias observed in the funnel plots, for both SBP and DBP.

With regard to DBP, certainty was further rated down for inconsistency and imprecision due to the large unexplained heterogeneity of results, and the wide variance in point estimates and CIs of included studies. While random effects model was applied and meta-analysis by subgroups was conducted for those studies measuring SBP, subgroup analysis could not be performed for DBP due to the small number of studies and their marked heterogeneity, but it should be borne in mind that AHT in children is mainly due to elevated SBP [41].

We are reasonably confident in not having missed any relevant studies, since we complemented the search with a manual review of the references of included studies. To the best of our knowledge, just two studies were excluded due to language (studies were written in Russian and Japanese) [65, 66]. Of note, papers assessing the effects of postnatal exposure on children’s or adolescents’ BP were not included due to the fact that they were all cross-sectional in nature [67–72].

## Conclusions

This study supports the hypothesis that maternal smoking in pregnancy could increase SBP of offspring during childhood and adolescence. Due to the low level of evidence, solid inferences cannot be drawn about the clinical relevance of these findings. Future cohorts should examine the effect of maternal smoking or ETS exposure on child’s SBP and DBP, after adjusting for different covariates.

## Supplementary Information

Below is the link to the electronic supplementary material.

Supplementary file1 (JPEG 66 KB)

Supplementary file2 (JPEG 22 KB)

Supplementary file3 (JPEG 35 KB)

Supplementary file4 (JPEG 13 KB)

Supplementary file5 (JPEG 572 KB)

Supplementary file6 (JPEG 611 KB)

Supplementary file7 (JPEG 590 KB)

Supplementary file8 (JPEG 573 KB)

Supplementary file9 (DOCX 20 KB)

Supplementary file10 (DOCX 19 KB)

Supplementary file11 (DOCX 32 KB)

Supplementary file12 (DOCX 20 KB)

Supplementary file13 (ODT 7 KB)

## Data Availability

All data generated or analyzed during this study are included in this published article (and its supplementary information files).

## Abbreviations

AHT: Arterial hypertension
BMI: Body mass index
BP: Blood pressure
CIs: Confidence intervals
DBP: Diastolic blood pressure
ETS: Environmental tobacco smoke
GRADE: Grading of recommendations, assessment, development, and evaluation
PRISMA: Preferred reporting items for systematic reviews and meta-analysis
SBP: Systolic blood pressure
