# Supplementary figures and images for: Maternal smoking in pregnancy and blood pressure during childhood and adolescence: a meta-analysis
Source: Eur J Pediatr. 2023 Feb 24;182(5):2119–32. doi: 10.1007/s00431-023-04836-1 (PMC10175379; doi:10.1007/s00431-023-04836-1)

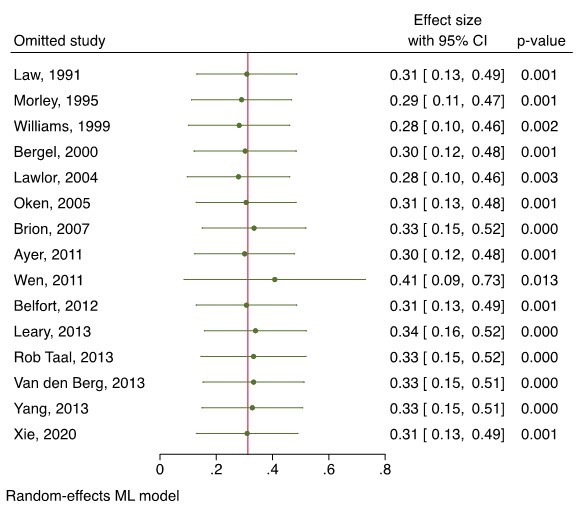

Supplement: Supplementary file 1 — Supplementary file1 (JPEG 66 KB) [file 431_2023_4836_MOESM1_ESM.jpeg]

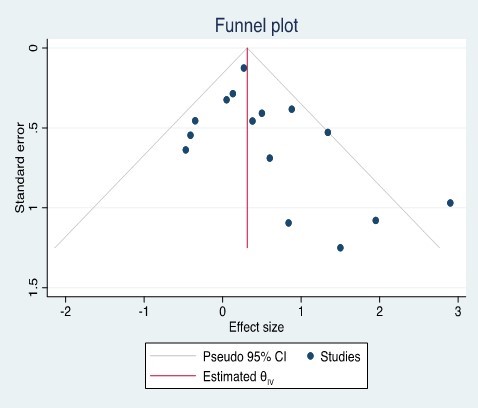

Supplement: Supplementary file 2 — Supplementary file2 (JPEG 22 KB) [file 431_2023_4836_MOESM2_ESM.jpeg]

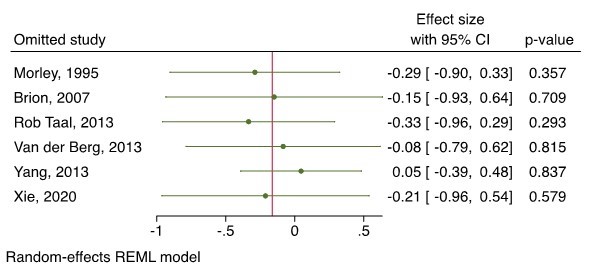

Supplement: Supplementary file 3 — Supplementary file3 (JPEG 35 KB) [file 431_2023_4836_MOESM3_ESM.jpeg]

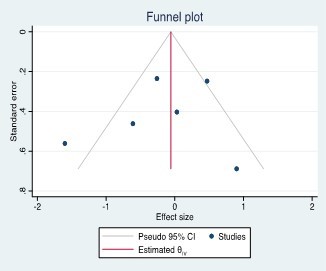

Supplement: Supplementary file 4 — Supplementary file4 (JPEG 13 KB) [file 431_2023_4836_MOESM4_ESM.jpeg]

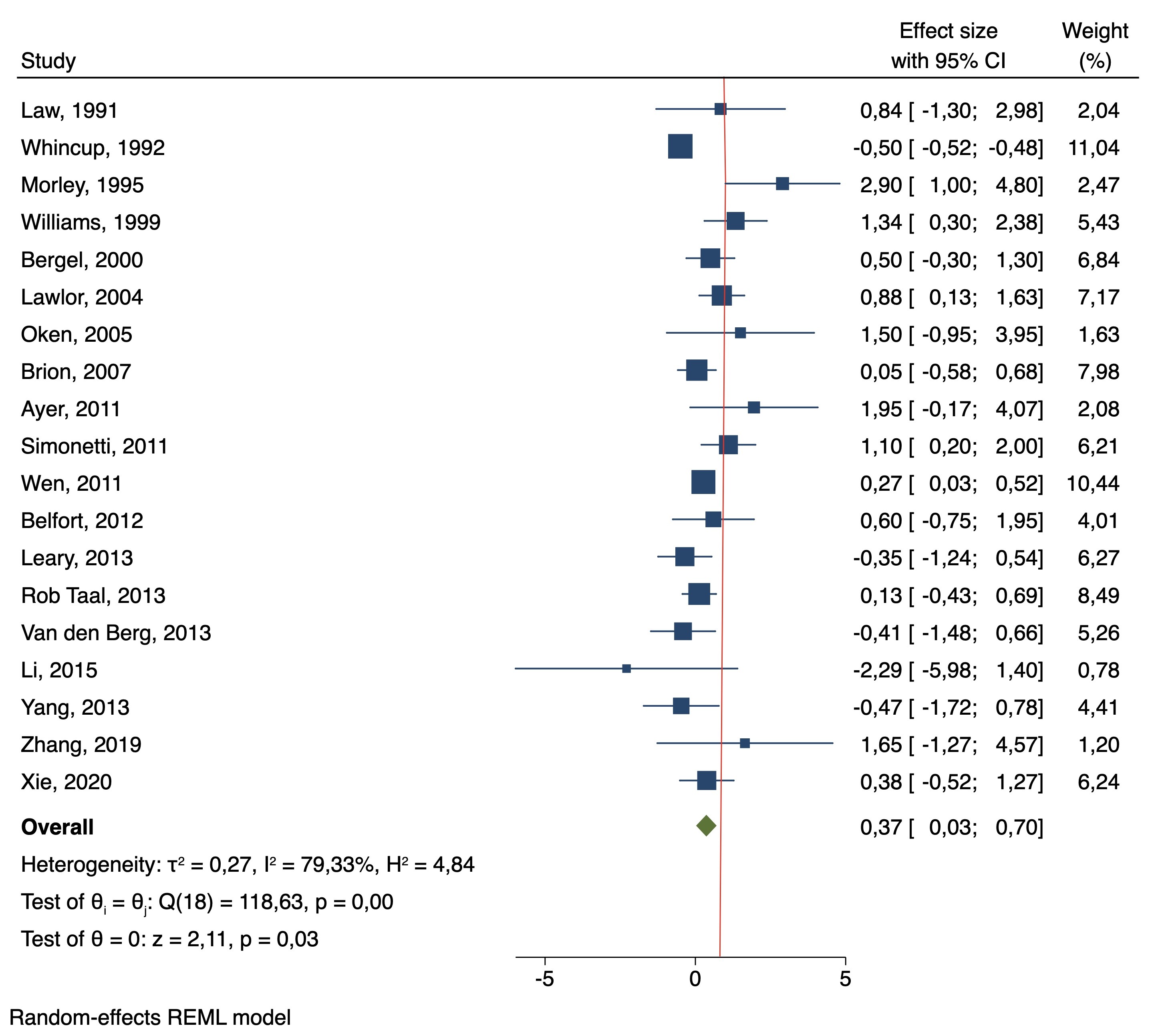

Supplement: Supplementary file 5 — Supplementary file5 (JPEG 572 KB) [file 431_2023_4836_MOESM5_ESM.jpeg]

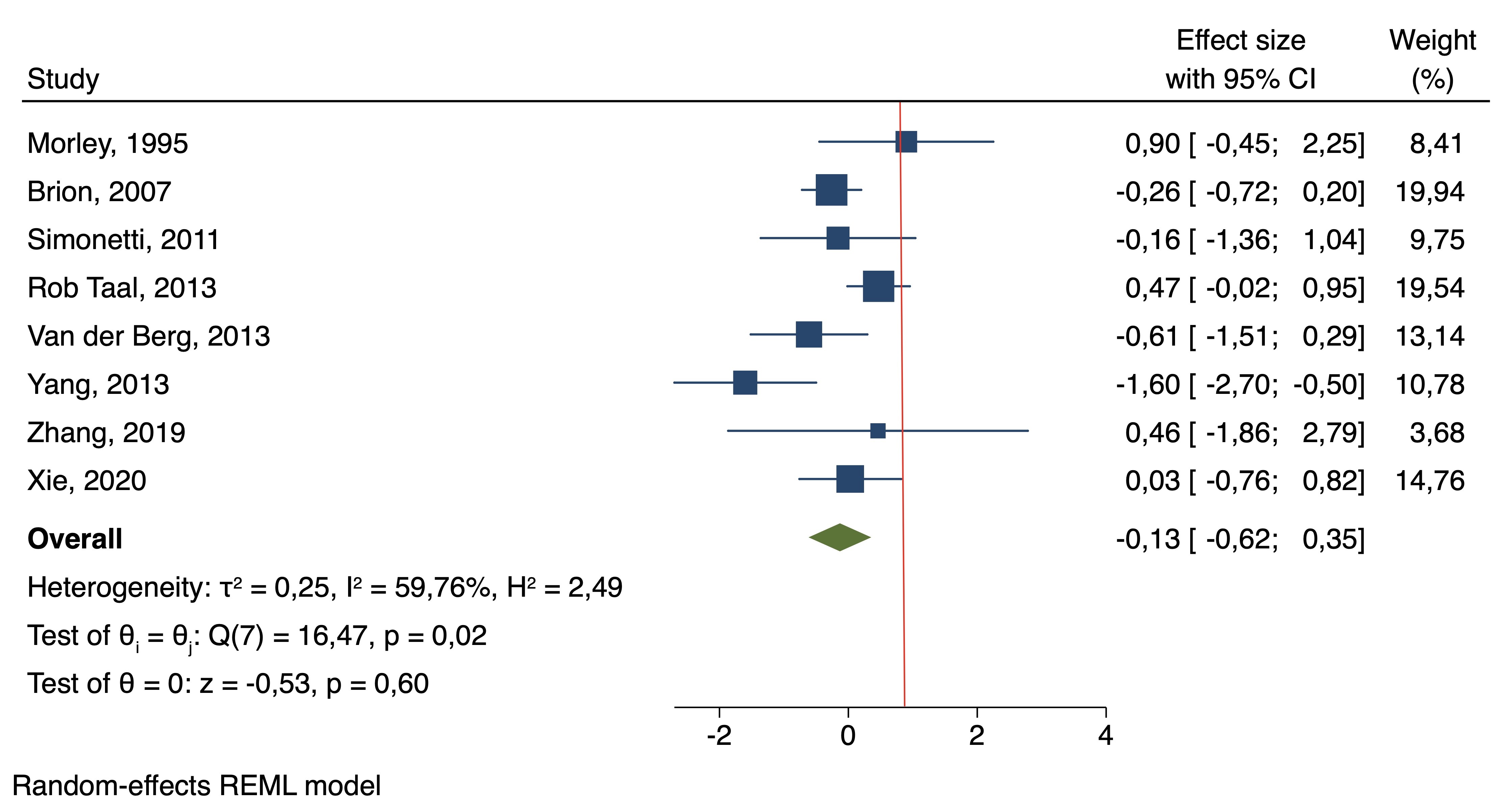

Supplement: Supplementary file 6 — Supplementary file6 (JPEG 611 KB) [file 431_2023_4836_MOESM6_ESM.jpeg]

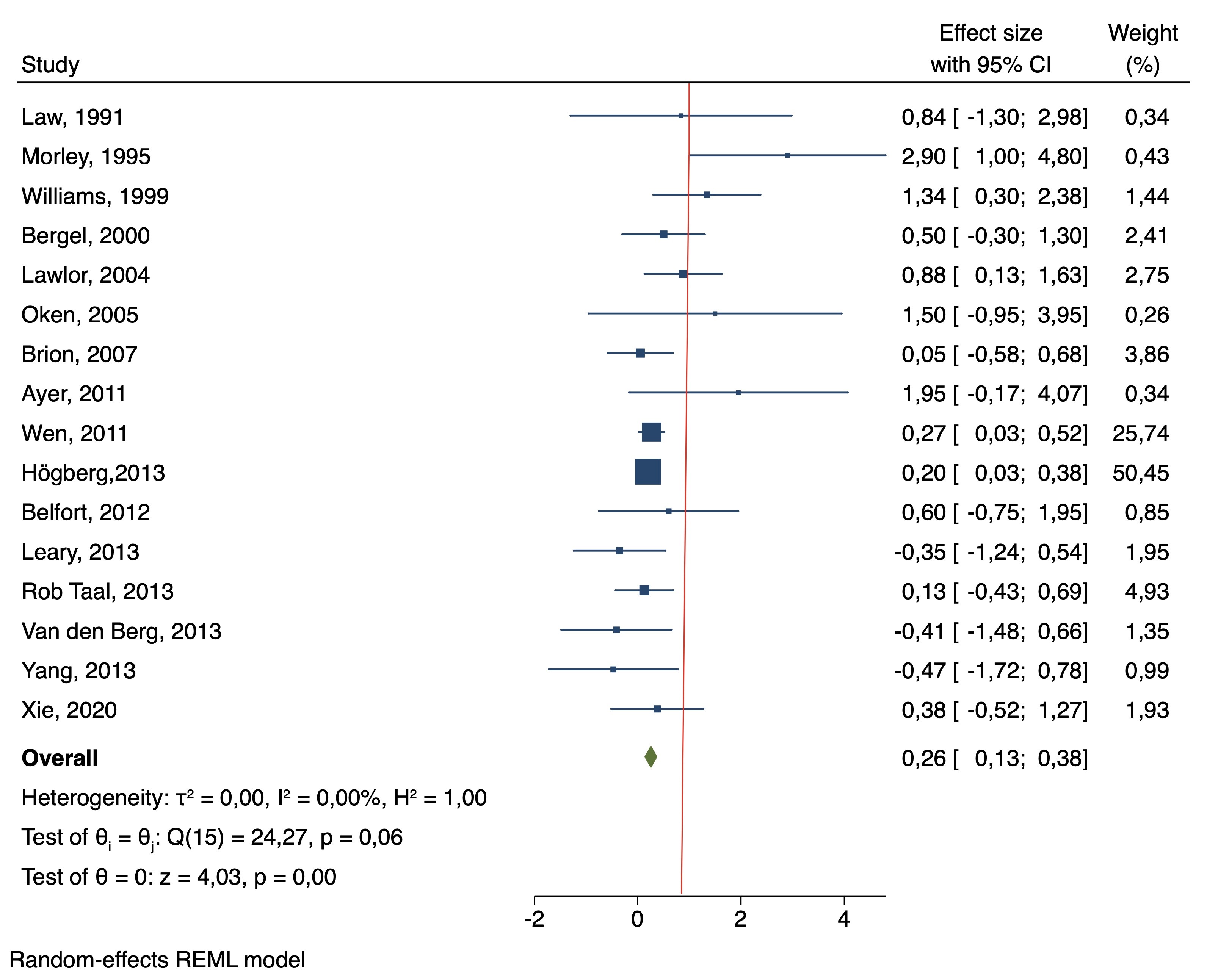

Supplement: Supplementary file 7 — Supplementary file7 (JPEG 590 KB) [file 431_2023_4836_MOESM7_ESM.jpeg]

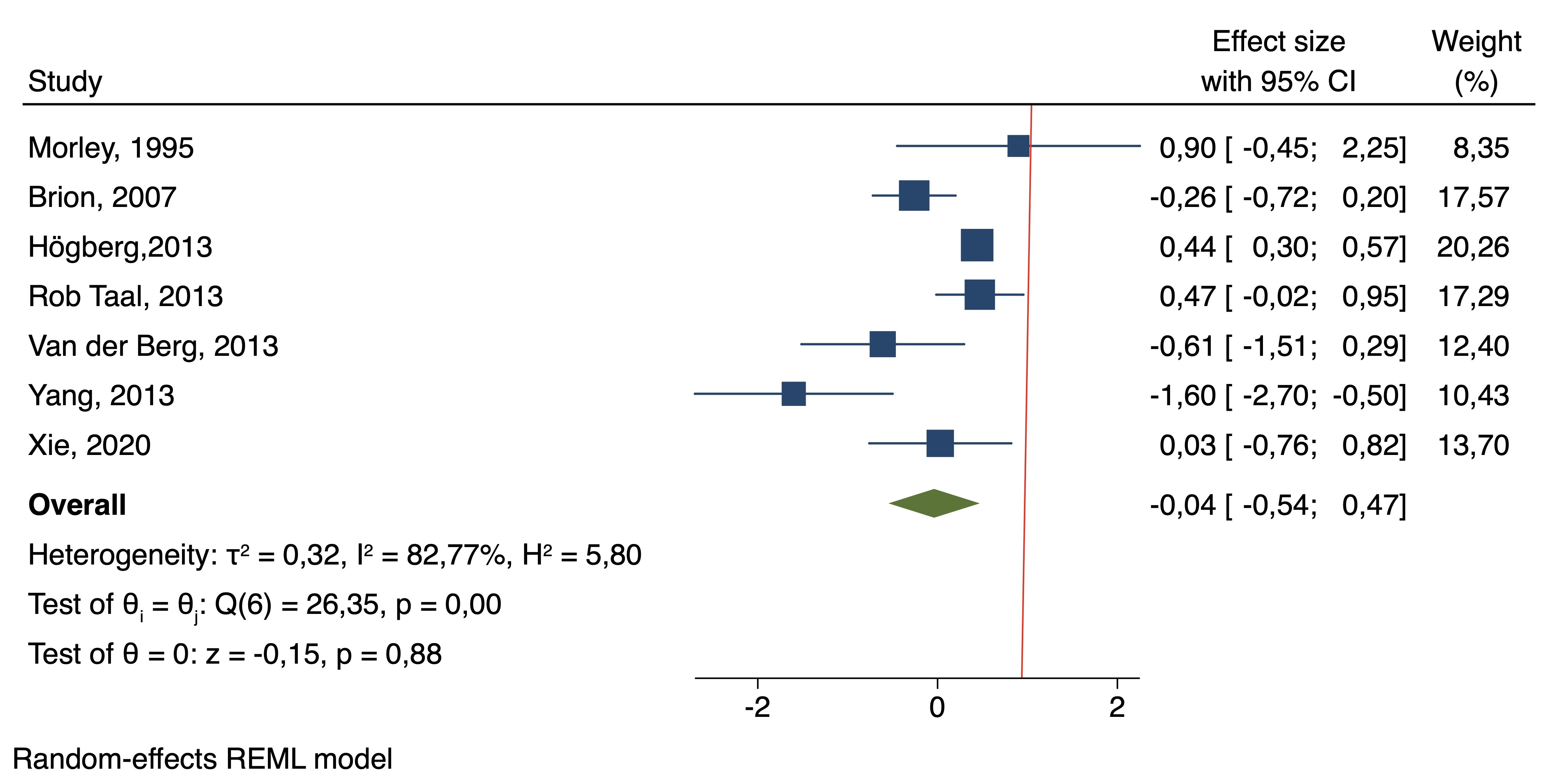

Supplement: Supplementary file 8 — Supplementary file8 (JPEG 573 KB) [file 431_2023_4836_MOESM8_ESM.jpeg]
