# Supplementary material for: Maternal smoking in pregnancy and blood pressure during childhood and adolescence: a meta-analysis
Source: Eur J Pediatr. 2023 Feb 24;182(5):2119–32. doi: 10.1007/s00431-023-04836-1 (PMC10175379; doi:10.1007/s00431-023-04836-1)
Supplement: Supplementary file 9 — Supplementary file9 (DOCX 20 KB) [file 431_2023_4836_MOESM9_ESM.docx]

**Supplementary Table 1.** Search query for each of the databases included

| **DATABASE** | **SEARCH QUERY** | **RECORDS** |
| --- | --- | --- |
| Medline (PubMed) | (("child"[MeSH Terms] OR "child"[All Fields] OR "children"[All Fields] OR "child s"[All Fields] OR "children s"[All Fields] OR "childrens"[All Fields] OR "childs"[All Fields] OR ("child"[MeSH Terms] OR "child"[All Fields] OR "children"[All Fields] OR "child s"[All Fields] OR "children s"[All Fields] OR "childrens"[All Fields] OR "childs"[All Fields]) OR "adolescences"[All Fields] OR "adolescency"[All Fields] OR "adolescent"[MeSH Terms] OR "adolescent"[All Fields] OR "adolescence"[All Fields] OR "adolescents"[All Fields] OR "adolescent s"[All Fields] OR ("adolescent"[MeSH Terms] OR "adolescent"[All Fields] OR "teen"[All Fields]) OR "adolescent"[MeSH Terms] OR "adolescent"[All Fields] OR "teenage"[All Fields] OR "teenager"[All Fields] OR "teenagers"[All Fields] OR "teenaged"[All Fields] OR "teenager s"[All Fields] OR "teenages"[All Fields]) AND ("blood pressure"[MeSH Terms] OR "blood pressure determination"[MeSH Terms] OR "arterial pressure"[MeSH Terms] OR "hypertension"[MeSH Terms] OR ("hypertense"[All Fields] OR "hypertension"[MeSH Terms] OR "hypertension"[All Fields] OR "hypertension s"[All Fields] OR "hypertensions"[All Fields] OR "hypertensive"[All Fields] OR "hypertensive s"[All Fields] OR "hypertensives"[All Fields]) OR "blood pressure"[All Fields] OR "systolic pressure"[All Fields] OR "diastolic pressure"[All Fields] OR "mean blood pressure"[All Fields])) AND ("tobacco smoke pollution"[MeSH Terms] OR ("tobacco"[All Fields] AND "smoke"[All Fields] AND "pollution"[All Fields]) OR "tobacco smoke pollution"[All Fields] OR ("secondhand"[All Fields] AND "smoke"[All Fields]) OR "secondhand smoke"[All Fields] OR ("tobacco smoke pollution"[MeSH Terms] OR ("tobacco"[All Fields] AND "smoke"[All Fields] AND "pollution"[All Fields]) OR "tobacco smoke pollution"[All Fields] OR ("environmental"[All Fields] AND "tobacco"[All Fields] AND "smoke"[All Fields]) OR "environmental tobacco smoke"[All Fields]) OR "tobacco smoke pollution"[MeSH Terms] OR ("tobacco"[MeSH Terms] OR "tobacco"[All Fields] OR "tobacco products"[MeSH Terms] OR ("tobacco"[All Fields] AND "products"[All Fields]) OR "tobacco products"[All Fields] OR "tobaccos"[All Fields] OR "tobacco s"[All Fields]) OR "smok*"[All Fields] OR ("maternal smoking"[All Fields] OR "paternal smoking"[All Fields])) Sort by: Most Recent | 6,495 |
| Embase | passive smoking/ or ("tobacco" and "smoke" and "pollution").af. or "tobacco smoke pollution".af. or ("secondhand" and "smoke").af. or "secondhand smoke".af. or ("environmental" and "tobacco" and "smoke").af. or "environmental tobacco smoke".af. or ("tobacco" or ("tobacco" and "products")).af. or tobacco/ or "tobacco products".af. or "tobaccos".af. or "tobacco s".af. Or "smok* ".af. or "maternal smoking".af. or "paternal smoking".af. And blood pressure/ or blood pressure measurement/ or arterial pressure/ or hypertension/ or "hypertense".af. or hypertension.af. or "hypertension s".af. or hypertensions.af. or hypertensive.af. or "hypertensive s".af. or "hypertensives".af. or ("blood pressure" or "systolic pressure" or "diastolic pressure" or "mean blood pressure").af. and ("child" or "children" or "child s" or "children s" or "childrens" or "childs" or "adolescences" or "adolescency" or "adolescent" or "adolescence" or "adolescents" or "adolescent s" or "teen" or "teenage" or "teenager" or "teenagers" or "teenaged" or "teenager s" or "teenages").af. | 5,113 |
| CENTRAL | #1 MeSH descriptor: [Tobacco Smoke Pollution] explode all trees  #2 ("passive smoking" OR ( "tobacco" AND "smoke" AND "pollution" ) OR "tobacco smoke pollution" OR ( "secondhand" AND "smoke" ) OR "secondhand smoke" OR ( "environmental" AND "tobacco" AND "smoke" ) OR "environmental tobacco smoke" OR ("tobacco" AND "products") OR tobacco OR "tobacco products" OR "tobaccos" OR "tobacco s" OR smok* OR "maternal smoking" OR "paternal smoking"):ti,ab,kw (Word variations have been searched)  #3 MeSH descriptor: [Tobacco] explode all trees  #4 #1 OR #2 OR #3  #5 MeSH descriptor: [Blood Pressure] explode all trees  #6 MeSH descriptor: [Blood Pressure Determination] explode all trees  #7 MeSH descriptor: [Hypertension] explode all trees  #8 MeSH descriptor: [Arterial Pressure] explode all trees  #9 ("hypertense" OR hypertension OR "hypertension s" OR hypertensions OR hypertensive OR "hypertensive s" OR "hypertensives" OR "blood pressure" or "systolic pressure" or "diastolic pressure" or "mean blood pressure"):ti,ab,kw (Word variations have been searched)  #10 #5 OR #6 OR #7 OR #8 OR #9  #11 ("child" OR "children" OR "child s" OR "children s" OR "childrens" OR "childs" OR "adolescences" OR "adolescency" OR "adolescent" OR "adolescence" OR "adolescents" OR "adolescent s" OR "teen" OR "teenage" OR "teenager" OR "teenagers" OR "teenaged" OR "teenager s" OR "teenages"):ti,ab,kw  #12 MeSH descriptor: [Child] explode all trees  #13 MeSH descriptor: [Adolescent] explode all trees  #14 MeSH descriptor: [Infant, Newborn] explode all trees  #15 #11 OR #12 OR #13 OR #14  #16 #4 AND #10 AND #15 in Trials | 427 |
