# Supplementary material for: Maternal smoking in pregnancy and blood pressure during childhood and adolescence: a meta-analysis
Source: Eur J Pediatr. 2023 Feb 24;182(5):2119–32. doi: 10.1007/s00431-023-04836-1 (PMC10175379; doi:10.1007/s00431-023-04836-1)
Supplement: Supplementary file 10 — Supplementary file10 (DOCX 19 KB) [file 431_2023_4836_MOESM10_ESM.docx]

| Representativeness of the sample (maximum 2*) | |  |
| --- | --- | --- |
|  | 1. No exclusion by characteristics or singleton delivery | ** |
|  | 1. Exclusion of high/low birth weight; cardiac/renal or other diseases; prematurity. | * |
|  | 1. Only preterm or low birth weight |  |
|  | 1. Definition of non-smoking mothers if their consumption is < 1cig/day |  |
|  |  |  |
| Sample size (maximum 1*) | |  |
|  | 1. Justified and satisfactory | * |
|  | 1. Not justified or not satisfactory |  |
|  |  |  |
| Ascertainment of the exposure (risk factor) (maximum 2*) | |  |
|  | 1. Cotinine and self-report | ** |
|  | 1. Self-reporting consumption during the whole pregnancy | * |
|  | 1. Self-reporting consumption only in a specific week or trimester of pregnancy |  |
|  | 1. Self-reporting at the time of measurement (possible recall bias) |  |
|  |  |  |
| Number of BP measurements (maximum 2*) | |  |
|  | 1. ≥ 3measurements | ** |
|  | 1. Two measurements | * |
|  | 1. One measurement or not specified |  |
|  |  |  |
| Adjustment variables (maximum 2*) | |  |
|  | 1. Maternal and children's anthropometric characteristics (BMI prior to-during pregnancy; weight gain during pregnancy; children's BMI at birth or at the time of BP measurement, fetal growth, small for gestational age)+ obstetric variables (parity, breastfeeding, gestational age)+ socioeconomic status+ maternal BP characteristics (history of AHT, pre-eclampsia, current AHT)+ children's characteristics (age, sex, lifestyles such as diet, exercise, time spent watching TV). | ** |
|  | 1. 3 of the above | * |
|  | 1. ≥2 of the above |  |
|  |  |  |
| Statistical test (maximum 1*) | |  |
|  | 1. The statistical test used to analyze the data is clearly described and appropriate, and the measurement of the association is presented, including confidence intervals and probability level (p-value). | * |
|  | 1. The statistical test is not appropriate, is not described or incomplete. |  |

**Supplementary Table 2**. Modified Newcastle-Ottawa quality assessment scale (items)

_BMI, body mass index; BP, blood pressure; AHT, arterial hypertension_
