# Supplementary material for: Maternal smoking in pregnancy and blood pressure during childhood and adolescence: a meta-analysis
Source: Eur J Pediatr. 2023 Feb 24;182(5):2119–32. doi: 10.1007/s00431-023-04836-1 (PMC10175379; doi:10.1007/s00431-023-04836-1)
Supplement: Supplementary file 11 — Supplementary file11 (DOCX 32 KB) [file 431_2023_4836_MOESM11_ESM.docx]

**Supplementary Table 3.** Studies excluded from the systematic review after screening and reading the full text (n= 34)

| AUTHOR | YEAR OF PUBLICATION | TITLE | | REASON FOR EXCLUSION | DATABASE | DOI/LINK |
| --- | --- | --- | --- | --- | --- | --- |
|  |  | |  |  |  |  |
| L S Webber | 1982 | | The interaction of cigarette smoking, oral contraceptive use, and cardiovascular risk factor variables in children: the Bogalusa Heart Study. | Not association measured (maternal smoking during pregnancy-offspring's BP during childhood/adolescence) | PubMed | https://doi.org/10.2105/ajph.72.3.266 |
| I B Tubol | 1989 | | Epidemiology of arteriosclerosis precursors in schoolchildren 10-13 years of age (data of a prospective study | Written in Russian | PubMed | <https://pubmed.ncbi.nlm.nih.gov/2733346/> |
| P H Whincup | 1992 | | Do maternal and intrauterine factors influence blood pressure in childhood? | Cross-sectional | PubMed | <https://doi.org/10.1136/adc.67.12.1423> |
| S A Hinchliffe | 1995 | | Maternal smoking and blood pressure in 7.5 to 8 year old offspring | Letter to editor | PubMed | <https://doi.org/10.1136/adc.73.4.378> |
| Stephen G Rostand | 2005 | | Racial disparities in the association of foetal growth retardation to childhood blood pressure | Lack of data (exposure period is unclear) | PubMed | <https://doi.org/10.1093/ndt/gfh833> |
| Seetha Shankaran | 2006 | | Fetal origin of childhood disease: intrauterine growth restriction in term infants and risk for hypertension at 6 years of age | Lack of data (risk of AHT, no SBP/DBP) | PubMed | <https://doi.org/10.1001/archpedi.160.9.977> |
| Caroline C Geerts | 2007 | | Tobacco smoke exposure of pregnant mothers and blood pressure in their newborns: results from the wheezing illnesses study Leidsche Rijn birth cohort | Infant population | PubMed | <https://doi.org/10.1161/hypertensionaha.107.091462> |
| S Viskari-Lähdeoja | 2008 | | Heart rate and blood pressure control in infants exposed to maternal cigarette smoking | Infant population | PubMed | <https://doi.org/10.1111/j.1651-2227.2008.00966.x> |
| J. Freihorst | 2011 | | Effect of parental smoking on children's blood pressure: Passive smoking is also a risk factor | Current tobacco smoke exposure (postnatal secondhand tobacco smoke) | Embase | http://dx.doi.org/10.1007/s00112-011-2397-3 |
| Giacomo D Simonetti | 2011 | | Determinants of blood pressure in preschool children: the role of parental smoking | Cross-sectional | PubMed | <https://doi.org/10.1161/circulationaha.110.958769> |
| Abolhassan Seyedzadeh | 2012 | | Relationship between Blood Pressure and Passive Smoking in Elementary School Children | Lack of data (exposure period is unclear) | PubMed | <https://www.ncbi.nlm.nih.gov/pmc/articles/PMC3564091/> |
| Lovisa Högberg | 2012 | | Effects of maternal smoking during pregnancy on offspring blood pressure in late adolescence | Only male population (older than 17 years old and not specified if smokers) | PubMed | <https://doi.org/10.1097/hjh.0b013e32835168f4> |
| Chi Le-Ha | 2013 | | Gender difference in the relationship between passive smoking exposure and HDL-cholesterol levels in late adolescence | Not association measured (maternal smoking during pregnancy-offspring's BP during childhood/adolescence) | PubMed | <https://doi.org/10.1210/jc.2013-1016> |
| Uri P Dior | 2014 | | Parental smoking during pregnancy and offspring cardio-metabolic risk factors at ages 17 and 32 | Inclusion of smoking adolescents | PubMed | <https://doi.org/10.1016/j.atherosclerosis.2014.05.937> |
| Luz Huntington-Moskos | 2014 | | Tobacco exposure, weight status, and elevated blood pressure in adolescents | Current tobacco smoke exposure (postnatal secondhand tobacco smoke) | PubMed | <https://doi.org/10.1007/s10900-014-9839-5> |
| Paula Azevedo Aranha Crispim | 2014 | | Risk factors associated with high blood pressure in two-to five-year-old children | Current tobacco smoke exposure (postnatal secondhand tobacco smoke) | PubMed | <https://doi.org/10.5935/abc.20130227> |
| G Cohen | 2014 | | Adverse circulatory effects of passive smoking during infancy: surprisingly strong, manifest early, easily avoided | Infant population | PubMed | <https://doi.org/10.1111/apa.12538> |
| Kay D Mann | 2015 | | Pathways between birth weight and later body size in predicting blood pressure: Australian Aboriginal Cohort Study 1987-2007 | Inclusion of smoking adolescents (older than 17 years old) | Pubmed | <https://doi.org/10.1097/hjh.0000000000000514> |
| Tsukasa Takemura | 2015 | | Childhood hypertension | Written in Japanese | PubMed | <https://pubmed.ncbi.nlm.nih.gov/26619664/> |
| L Li | 2015 | | Maternal smoking in pregnancy association with childhood adiposity and blood pressure | Cross-sectional | PubMed | <https://doi.org/10.1111/ijpo.12046> |
| P Brambilla | 2016 | | Predictors of blood pressure at 7-13 years: The "new millennium baby" study | Lack of data (exposure period is unclear) | PubMed | <https://doi.org/10.1016/j.numecd.2015.11.005> |
| Emmanuella Magriplis | 2017 | | Maternal smoking and risk of obesity in school children: Investigating early life theory from the GRECO study | Lack of data (not mean-adjusted BP) and cross-sectional | PubMed | <https://doi.org/10.1016/j.pmedr.2017.10.001> |
| Judith A Groner | 2017 | | Secondhand Smoke Exposure and Preclinical Markers of Cardiovascular Risk in Toddlers | Lack of data (exposure period is unclear) | PubMed | <https://doi.org/10.1016/j.jpeds.2017.06.032> |
| Stevens | 2018 | | In utero exposure to tobacco smoke, subsequent cardiometabolic risks, and metabolic syndrome among U.S. adolescents | Lack of data (no mean-adjusted differences in BP) and cross-sectional and | PubMed | <https://doi.org/10.1016/j.annepidem.2018.06.010> |
| Maria Cabral | 2018 | | Maternal Smoking: A Life Course Blood Pressure Determinant? | Lack of data (no mean-adjusted differences in BP) | PubMed | <https://doi.org/10.1093/ntr/ntx117> |
| Maria Adriana Cornelia Jansen | 2019 | | Pre-pregnancy parental BMI and offspring blood pressure in infancy | Not association measured (maternal smoking during pregnancy-offspring's BP during childhood/adolescence) | CENTRAL | <https://doi.org/10.1177/20474873198581> |
| Marietta Charakida | 2019 | | Early vascular damage from smoking and alcohol in teenage years: the ALSPAC study | Not association measured (maternal smoking during pregnancy-offspring's BP during childhood/adolescence) | PubMed | <https://doi.org/10.1093/eurheartj/ehy524> |
| J J A De Smidt | 2019 | | In utero teratogen exposure and cardiometabolic risk in 5-year-old children: a prospective pediatric study | Not association measured (maternal smoking during pregnancy-offspring's BP during childhood/adolescence) | PubMed | <https://doi.org/10.1080/14767058.2019.1692337> |
| Haishan Zhang | 2019 | | In utero and postnatal exposure to environmental tobacco smoke, blood pressure, and hypertension in children: the Seven Northeastern Cities study | Cross-sectional | PubMed | <https://doi.org/10.1080/09603123.2019.1612043> |
| Felicia Nordenstam | 2019 | | Blood Pressure and Heart Rate Variability in Preschool Children Exposed to Smokeless Tobacco in Fetal Life | Other tobacco products (Swedish SNUS) | PubMed | <https://doi.org/10.1161/jaha.119.012629> |
| Shu-Li Xu | 2020 | | Pet ownership in utero and in childhood decreases the effects of environmental tobacco smoke exposure on hypertension in children: A large population based cohort study | Not association measured (maternal smoking during pregnancy-offspring's BP during childhood/adolescence) | PubMed | <https://doi.org/10.1016/j.scitotenv.2020.136859> |
| Marleen Hamoen | 2020 | | Development of a prediction model to target screening for high blood pressure in children | Current tobacco smoke exposure (postnatal secondhand tobacco smoke) | Pubmed | <https://doi.org/10.1016/j.ypmed.2020.105997> |
| Yanhui Li | 2020 | | Association Between Maternal Lifestyle and Risk of Metabolic Syndrome in Offspring-A Cross-Sectional Study From China | Current tobacco smoke exposure (postnatal secondhand tobacco smoke) | PubMed | <https://doi.org/10.3389/fendo.2020.552054> |
| Seyram Kaali | 2021 | | Prenatal Household Air Pollution Exposure, Cord Blood Mononuclear Cell Telomere Length and Age Four Blood Pressure: Evidence from a Ghanaian Pregnancy Cohort | Not association measured (maternal smoking during pregnancy-offspring's BP during childhood/adolescence) | CENTRAL | <https://doi.org/10.3390/toxics9070169> |
