# Supplementary material for: Maternal smoking in pregnancy and blood pressure during childhood and adolescence: a meta-analysis
Source: Eur J Pediatr. 2023 Feb 24;182(5):2119–32. doi: 10.1007/s00431-023-04836-1 (PMC10175379; doi:10.1007/s00431-023-04836-1)
Supplement: Supplementary file 12 — Supplementary file12 (DOCX 20 KB) [file 431_2023_4836_MOESM12_ESM.docx]

| **Author, year** | **Representativeness of the sample** | **Sample size** | **Ascertainment of the exposure** | | **Blood pressure measurements** | **Adjustement for covariates** | **Statistical test** | **Score** |
| --- | --- | --- | --- | --- | --- | --- | --- | --- |
| Law, 1992 | * | * |  | ** | |  | * | 4 |
| Morley, 1995 |  |  |  | ** | |  | * | 3 |
| Williams, 1999 | ** |  |  | ** | |  |  | 4 |
| Bergel, 2000 | ** |  | * | ** | |  |  | 5 |
| Blake*, 2000 | ** |  | * | * | |  | * | 5 |
| Lawlor, 2004 | ** | * | * | * | |  | * | 6 |
| Okens, 2005 | ** |  |  | ** | | * | * | 6 |
| Brion, 2007 | ** | * | * | * | | * | * | 7 |
| Laura*, 2010 | ** | * |  | * | |  | * | 5 |
| Ayer, 2011 | * | * | * | ** | |  | * | 5 |
| Wen, 2011 | * | * | ** |  | | * | * | 5 |
| Belfort, 2012 |  |  | * | ** | |  |  | 3 |
| Leary, 2013 | ** | * | * | * | | * | * | 7 |
| Rob Taal, 2013 | ** | * | * | ** | | ** | * | 9 |
| Van der Berg, 2013 | * | * |  | ** | | ** | * | 7 |
| Rauschert*, 2019 | ** |  |  |  | |  | * | 3 |
| Yang, 2013 | * | * | * | * | |  |  | 4 |
| Xie, 2020 | ** | * |  | ** | | ** | * | 8 |
| De Smidt*, 2020 | * |  |  | ** | |  | * | 4 |
| Grouleff*, 2021 | ** |  | ** | * | |  | * | 6 |
| Cajachagua-Torres*, 2021 | ** | * | * | ** | | ** | * | 9 |

**Supplementary Table 4**. Quality of the 21 studies included in the systematic review (Modified Newcastle-Ottawa scale)

^*Studies excluded from the meta-analysis^
