# Supplementary material for: Maternal smoking in pregnancy and blood pressure during childhood and adolescence: a meta-analysis
Source: Eur J Pediatr. 2023 Feb 24;182(5):2119–32. doi: 10.1007/s00431-023-04836-1 (PMC10175379; doi:10.1007/s00431-023-04836-1)
Supplement: Supplementary file 6 [file 431_2023_4836_MOESM13_ESM.odt]

Supplementary Table 5. GRADE evidence profile

| Number of studies | Study design | Risk of bias | Inconsistency | Uncertainty or lack of direct evidence | Inaccuracy | Publication bias | Other considerations | Quality |
| --- | --- | --- | --- | --- | --- | --- | --- | --- |
| Systolic blood pressure |
| 15 (SBP) | Cohort studies | Serious limitations1 | No serious limitations | No serious limitations | No serious limitations | Detected | - | Low |
| 6 (DBP) | Cohort studies | Serious limitations1 | Serious limitations | No serious limitations | Serious limitations | Detected | - | Very low |

1 Classification, selection, information, detection, confounding, observer bias, etc.
